# In vitro degradation and cell attachment studies of a new electrospun polymeric tubular graft

**DOI:** 10.1007/s40204-015-0038-y

**Published:** 2015-04-09

**Authors:** Harsh N. Patel, Kevin N. Thai, Sami Chowdhury, Raj Singh, Yogesh K. Vohra, Vinoy Thomas

**Affiliations:** 1 Department of Biomedical Engineering, University of Alabama at Birmingham (UAB), Birmingham, AL 35294 USA; 2 Department of Materials Science and Engineering, University of Alabama at Birmingham (UAB), Birmingham, AL 35294 USA; 3 Vivo Biosciences Inc, Birmingham, AL 35205 USA; 4 Center for Nanoscale Materials and Biointegration (CNMB), University of Alabama at Birmingham (UAB), Birmingham, AL 35294 USA

**Keywords:** Poliglecaprone, Lipase, Electrospinning, Mechanical properties, Vascular graft

## Abstract

Electrospinning technique was utilized to engineer a small-diameter (id = 4 mm) tubular graft. The tubular graft was made from biocompatible and biodegradable polymers polycaprolactone (PCL) and poliglecaprone with 3:1 (PCL:PGC) ratio. Enzymatic degradation effect on the mechanical properties and fiber morphology in the presence of lipase enzyme were observed. Significant changes in tensile strength (1.86–1.49 MPa) and strain (245–205 %) were noticed after 1 month in vitro degradation. The fiber breakage was clearly evident through scanning electron microscopy (SEM) after 4 weeks in vitro degradation. Then, the graft was coated with a collagenous protein matrix to impart bioactivity. Human umbilical vein endothelial cells (HUVECs) and aortic artery smooth muscle cells (AoSMCs) attachment on the coated graft were observed in static condition. Further, HUVECs were seeded on the lumen surface of the grafts and exposed to laminar shear stress for 12 h to understand the cell attachment. The coated graft was aged in PBS solution (pH 7.3) at 37 °C for 1 month to understand the coating stability. Differential scanning calorimetry (DSC) and Fourier transform infrared spectroscopy (FTIR) suggested the erosion of the protein matrix from the coated graft under in vitro condition.

## Introduction

Approximately, 800,000 coronary artery bypass graft surgeries (CABG) are performed worldwide each year (Dahl et al. 2011). Alarmingly, one-third of the patients who undergo the CABG procedure do not have viable autologous grafts (Wang et al. 2007). Further, there are 202 million people suffering from peripheral artery disease (PAD), and particularly in the USA ~8.5 million Americans aged ≥40 years have PAD (Go et al. 2014; Fowkes et al. 2013). Surgeons have used non-degradable synthetic polytetrafluoroethylene (PTFE) or Dacron medium- to large-diameter grafts which provide 10 years of symptom-free lifestyle; however they have extremely poor performance due to thrombotic occlusion and intima hyperplasia when their diameter is <6 mm (Song et al. 2011; Clowes et al. 1985). In the pediatric population, non-degradable prosthetic grafts are unable to match with somatic growth, resulting in requiring a second or third surgery (Cittadella et al. 2013). Therefore, there is a desperate need for a small-diameter (<6 mm) vascular graft which can replace and or repair the damaged native blood vessel, promote regeneration of a neo tissue, and completely be bioabsorbed in the long run.

Researchers have tried various approaches to engineer such an ideal vascular graft, for instance, natural scaffolds (collagen, elastin, fibrin), self-assembled cell sheets, synthetic scaffolds, and decellularized matrix (allogenic, xenogenic, heterogenic) (Seifu et al. 2013). However, long fabrication time, potential transmission of disease, thrombus formation, hyperplasia, and immune rejections are critical issues associated with these approaches (Seifu et al. 2013). Hence, a general movement was started to engineer coated grafts by combining biodegradable and biocompatible synthetic polymers with natural proteins such as collagen, elastin, and fibrin (Sell et al. 2009). Examples of biodegradable synthetic polymers used in vascular tissue engineering applications are poly(lactide acid) PLA, polycaprolactone (PCL), polyurethanes (PU), polyglycolic acid (PGA), and polydioxanone (PDO) (Sell et al. 2009; Boland et al. 2005; Stitzel et al. 2001; Kidoaki et al. 2005; Lee et al. 2008a). In this approach, a synthetic polymer will provide mechanical integrity and natural proteins provide biocompatibility and extracellular matrix (ECM)-mimicking environment for better cell attachment and proliferation.

To fabricate scaffolds for tissue engineering application, researchers have explored many techniques such as solvent casting, phase separation, fiber self-assembly, electrospinning, melt molding, decellularization, gas foaming, and laser sintering (Song et al. 2011). However, the electrospinning technique has gained particular interest due to its simplicity and versatility. This method is capable of forming nano–microscale fibers which can mimic the natural tissue ECM morphology; hence, scaffolds made from this technique can be utilized for various biomedical applications such as drug delivery and soft/hard tissue regeneration (Liu et al. 2013). The electrospun biodegradable scaffolds have a porous structure to allow cell migration and infiltration, higher surface area for better cell attachment, and a tunable degradation rate which is necessary to promote neo-tissue ingrowth (Sell et al. 2010).

A new tubular graft from biocompatible and biodegradable PCL and poliglecaprone (PGC) polymers was fabricated by an electrospinning technique (Patel et al. 2015). PCL is a semicrystalline polymer which provides a slow degradation time (~2 years); therefore, it can play a critical role in tissue engineering application when a scaffold requires a longer time to support the damage tissue and promote regeneration (Gunatillake and Adhikari 2003). PCL has great viscoelastic properties, which provide the key qualities for vascular tissue engineering application (Lee et al. 2003). De Valence et al. (2012) showed a great structural integrity for the graft made from PCL in rat abdominal aorta model. Unlike PGA, PLA, and poly-lactide-co-glycolic acid (PLGA), PCL does not undergo plastic deformation and failure when exposed to long cyclic strain; therefore, it can be an excellent and critical component in vascular graft application (Lu et al. 2008). PGC in the form of Monocryl^®^ monofilament sutures displayed excellent tensile properties and 20–30 % reduction in strength after 2 weeks in vivo (Bezwada et al. 1995). Complete absorption of PGC in human body between 90 and 120 days with slight to minimal tissue reaction has been confirmed (Bezwada et al. 1995). Therefore, a blend of PCL and PGC can provide the required mechanical integrity, and faster PGC degradation can provide room for neo-tissue formation.

Various biodegradable polymers and natural polymers were combined to engineer an ideal vascular graft in the past. He et al. (2009) combined PLLA with PCL and coated with collagen to create a small-diameter vascular graft and noticed promising biocompatibility and in vivo results. Ju et al. (2010) used PCL with collagen to fabricate a tubular graft and showed endothelial cell (ECs) adhesion as well as smooth muscle cell (SMCs) infiltration from the outer surface to the lumen side. Han et al. (2010) co-electrospun a blend of PLGA, gelatin, and elastin to create a scaffold for vascular tissue engineering and studied EC and SMC attachment. Pandis et al. (2010) fabricated a hyaluronan-based scaffold and examined its in vivo performance in rats. Pankajkshan et al. (2008) coated PCL scaffold with fibrin to engineer a potential vascular graft and observed EC lining in 15 and 30 days after cultured. He et al. (2011) utilized PLLA and PCL with fibrinogen for potential soft tissue engineering applications as well. Interestingly, Wang et al. (2009) combined PLA with silk fibroin to generate tubular scaffolds and examined mechanical as well as biocompatibility with different cell lines for potential use in blood vessel tissue engineering application. Though extraordinary efforts have been undertaken to solve the critical need for an ideal vascular graft, a clinically available bio-hybrid tubular graft requires further research. In our recent publication, we have shown that 3:1 (PCL:PGC) blend had the most desirable mechanical properties for vascular graft application (Patel et al. 2015). PCL breaks down by hydrolytic degradation mechanism, and it has been studied by Pena et al. (2006). However, there have been studies showing that the hydrolytic degradation may be catalyzed by enzymes such as lipase (Zeng et al. 2004; Gan et al. 1999). Lipase, an extracellular hydrolytic enzyme which is water soluble, is able to digest aliphatic polyesters such as PCL (Rizzarelli et al. 2004; Tokiwa and Suzuki 1977). Researchers have incorporated collagen, elastin, chitosan, hyaluronic acid, fibrin, and gelatin to create bioactive scaffold for vascular tissue engineering application (McClure et al. 2009a, b; Pankajakshan et al. 2008; Yin et al. 2013; He et al. 2005; Antunes et al. 2010; Thomas et al. 2007; Zhang et al. 2009). However, a blood vessel is composed of three distinct layers which have different proteins that work synergistically to provide proper functionality (Stegemann et al. 2007). Hence, it may be beneficial to incorporate a collagenous matrix which is made of different proteins such as various types of collagen and laminin to closely mimic the structure of a blood vessel. Hence, to make our graft’s surface bioactive for cell adhesion, the graft was coated with a collagenous matrix (Siegal and Singh 2010) by dip coating. Finally, AoSMCs and HUVECs attachment was studied on the coated graft in static condition. Further, shear stress plays a crucial role in the long-term maintenance of blood vessel functionality (Traub and Berk 1998). Hence, HUVECs attachment was studied under a dynamic condition.

## Materials and methods

### Materials

Polycaprolactone (PCL) with inherent viscosity between 1.0 and 1.3 dL/g in CHCl_3_ was obtained from LACTEL Absorbable Polymers (Birmingham, AL). Poliglecaprone (PGC) was acquired in the form of absorbable surgical sutures under the trade name of Monocryl^®^ (Ethicon). The solvent 1,1,1,3,3,3-hexafluoro-2-propanol (HFP) was purchased from Sigma-Aldrich (St. Louis, MO) to dissolve PCL and PGC (weight ratio PCL:PGC = 3:1) and make a homogeneous solution. Lipase (*Pseudomonas fluorescens*) was purchased from Sigma-Aldrich. The protein matrix (HB) was provided by Vivo Biosciences Inc. (Birmingham, AL). HUVECs and AoSMCs (Lonza Group Ltd) were kindly provided by Dr. Jun’s Laboratory at passage 3. The HUVECs were cultured into the endothelial cell growth media (EGM-2 Lonza Group Ltd) at 37 °C under 5 % CO_2_. The AoSMCs were cultured into the smooth muscle cells growth media (SMGM-2 Lonza Group Ltd) at 37 °C under 5 % CO_2_.

### Fabrication of electrospun tubular graft

Tubular electrospun graft was engineered by the following method described in our recent publication (Patel et al. 2015). Briefly, PLC and PGC were mixed at a ratio of 75:25 (wt%) and a 12 % (w/v) solution was obtained. The electrospinning technique was utilized to produce the graft. Then, the solution was loaded into a BD 3 mL syringe with a 25 gauge needle (Small Parts Inc). A high-voltage power supply (M826, Gamma High-Voltage Research, Ormond Beach, FL) was connected to the needle. The infusion rate (1 mL/h), voltage (15 kV), and the distance between the needle tip and the mandrel (25 cm) were used to produce fine nano–microscale fibers. The fibers were collected onto a 4 mm-diameter 303 stainless steel mandrel, rotating at 400 rpm. The tubular graft was removed from the mandrel and put in a desiccator for 24 h to remove the solvent residuals.

### In vitro enzymatic degradation

To understand the lipase enzyme effect on the mechanical properties, the graft was exposed to the PBS solution with lipase concentration of 2.5 μg/mL for 4 weeks. Twice a week, the lipase solution was changed. Samples were prepared and tested at 2 and 4 weeks’ time periods to obtain changes in mechanical properties due to enzymatic degradation. Tensile specimens (*n* = 6) were prepared by cutting the scaffolds into rectangular stripes (3 mm × 10 mm) in accordance with ASTM standard D882. A dynamic mechanical analyzer with tensile fixture (DMA, TA instruments) was used. The samples were mounted first on the fixture. The samples were tested uniaxially using 18 N load cell at a ramp 0.1 N mm^−1^. All values such as elastic modulus, percent elongation to failure, and ultimate tensile strength were obtained from stress–strain curves generated by the TA instrument software. Further, FE-SEM (Quanta FEG 650 from FEI, Hilsboro, OR) was utilized to understand the morphology of the vascular graft before and after enzymatic degradation, and grafts were cut and sputter-coated with Au–Pd to understand the surface morphology. To obtain mass loss (%) data, samples [1 cm × 1 cm (*n* = 6)] were aged under enzymatic solution at 37 °C for 4 weeks (Patel et al. 2015). At the second and fourth week’s interval, the samples were removed from the solution and placed under vacuum at room temperature and the mass loss (%) was determined.

### Protein matrix-coated tubular graft under in vitro condition

The tubular graft was coated with a collagenous protein matrix by dip coating. The tubular graft was soaked in the PBS solution (pH 7.3) overnight at 4 °C to wet the surface for better collagenous protein matrix absorption. Then, the tubular graft was dip coated by soaking in the collagenous protein matrix with higher density (3 mg/mL) on the outside (pH 7.3) for 2 h at 4 °C. Then, the inner layer was coated with lower density (1 mg/mL) of collagenous matrix (pH 7.3) for 2 h at 4 °C. Finally, the coated graft was placed in the incubator at 37 °C in a humid environment for the gelation and stabilization of the collagenous protein matrix for 2 h. FT-IR and differential scanning calorimetric (DSC) techniques were utilized to confirm the presence of the protein matrix on the graft before and after aging in physiological media. The Bruker alpha FTIR spectrometer was used with ATR mode to acquire absorbance spectrum (64 scans per sample, ranging from 4000 to 400 cm^−1^) for coated graft. Samples were tested by utilizing a DSC instrument (TA Instruments Q100) from −75 to 250 °C at a rate of 10 °C/min.

### HUVECs and AoSMCs attachment to coated graft

HUVECs and AoSMCs (Lonza Group Ltd) were kindly provided by the Dr. Jun’s Laboratory at UAB at passage 3. The tubular scaffolds were sterilized and preconditioned before cell seeding by ethanol and UV sterilization. The scaffolds were placed in 48-well plates and incubated with 100 % fetal bovine serum (FBS) (VWR international) at 37 °C for 4 h (Zhang et al. 2010). Finally, the FBS was taken out from the well. HUVECs were seeded on the scaffold with 6 × 10^5^ cells/cm^2^ density. AoSMCs were seeded on the scaffolds with 1 × 10^4^ cells/cm^2^ density. The morphology of the HUCECs and AoSMCs was observed. The scaffolds were stained with DAPI (Sigma-Aldrich) and rhodamine phalloidin (Sigma-Aldrich) staining to stain nucleus and actin filaments respectively. Nikon A1 confocal camera was used to acquire all the images at 40× magnification.

### HUVECs attachment under laminar shear stress

The dynamic flow chamber system was set up as shown in Fig. 7. The circular flow chamber kit was (GlycoTech Inc) purchased. The circular flow chamber was set up by following the instructions provided by the manufacturer. The silicon rubber gasket with flow width 1.00 cm and thickness 0.010 inch was used. The peristaltic pump (Fisher Scientific™) was used to create a constant laminar flow. The HUVECs seeded scaffold was carefully placed into the parallel-flow chamber. The gas exchange chamber was also placed to have proper CO_2_ gas exchange. The entire system was placed in the sterilized incubator with humid condition, 37 °C temperature, and proper supply of CO_2_. The cell-seeded scaffolds were exposed to this laminar flow condition for 12 h to understand the cell’s adhesion strength (Savoji et al. 2014; Gigout et al. 2011). The flow rate was set up to expose scaffolds to similar shear stress as in the physiological condition (Malek et al. 1999; Dela Paz and D’Amore 2009). The number of HUVECs retained on the scaffold exposed to the laminar flow was compared with non-exposed HUVECs-seeded scaffold.

## Results and discussion

**Fig. 1:**
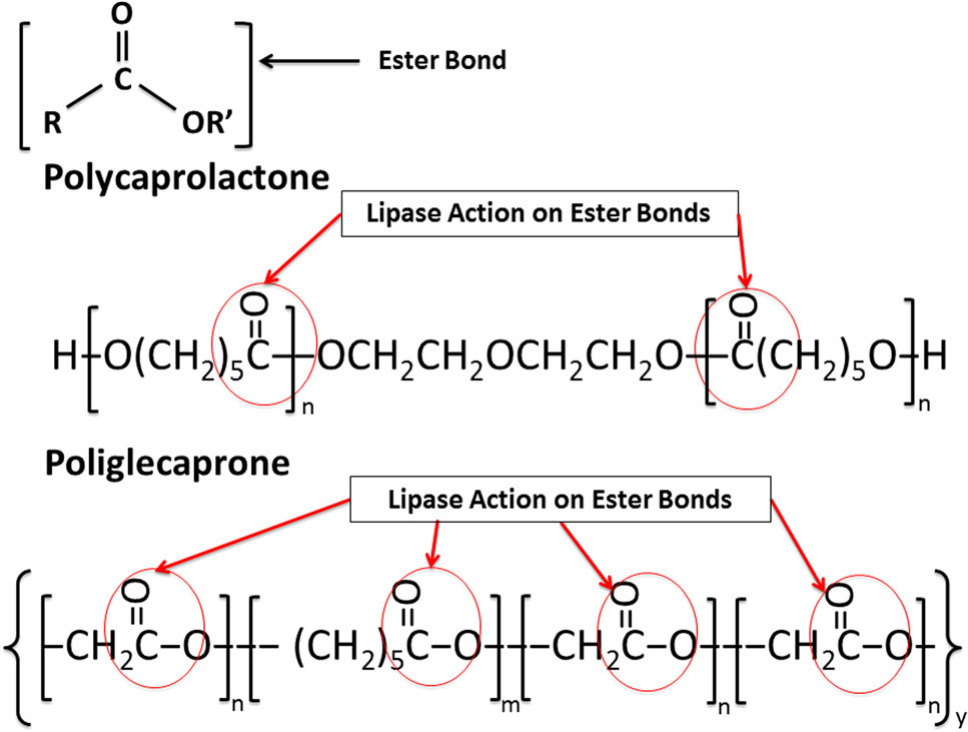
Lipase effect on polycaprolactone and poliglecaprone polymers

Our tubular graft engineered from 3:1 (PCL:PGC) by utilizing an electrospinning technique was made of nano–microscale fibers (diameter ranges from 0.5 to 1.0 μm). Its chemical composition had a major amount of the PCL component; hence, it was important for us to understand the effect of the enzyme on the mechanical properties. Therefore, we exposed the electrospun graft to the lipase-containing PBS solution for 1 month to observe changes in mechanical properties. The hydrolytic degradation of PCL and PGC occurs due to the breakage of the ester bonds as shown in the Fig. 1. The lipase enzyme also attacks these ester bonds as shown in Fig. 1, to break the long polymer chain into smaller oligomers which are soluble in water (Rizzarelli et al. 2004).

**Fig. 2:**
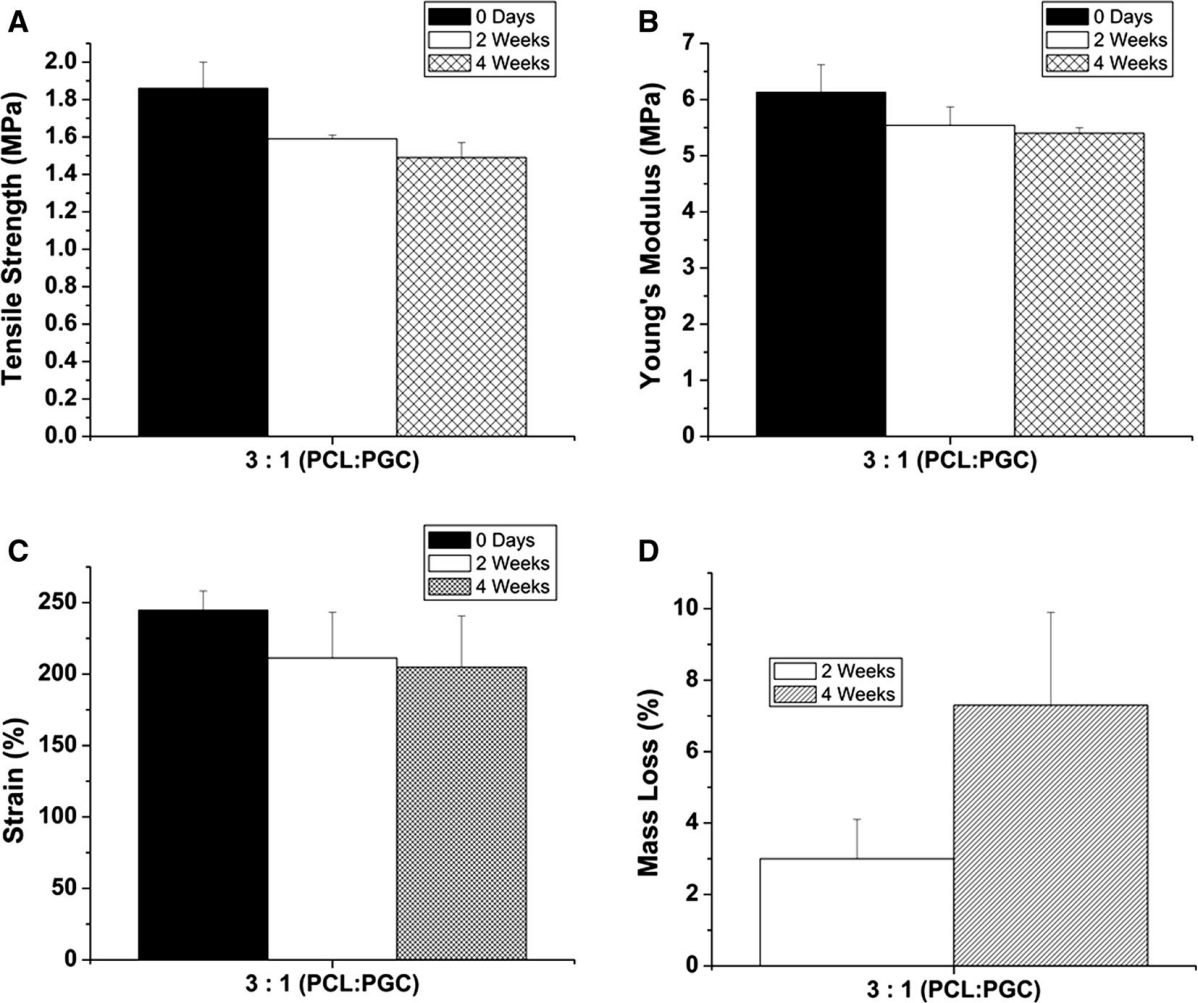
Lipase degradation effect on mechanical properties of graft **a** tensile strength, **b** modulus of elasticity, **c** strain (%), and **d** mass loss (%)

**Fig. 3:**
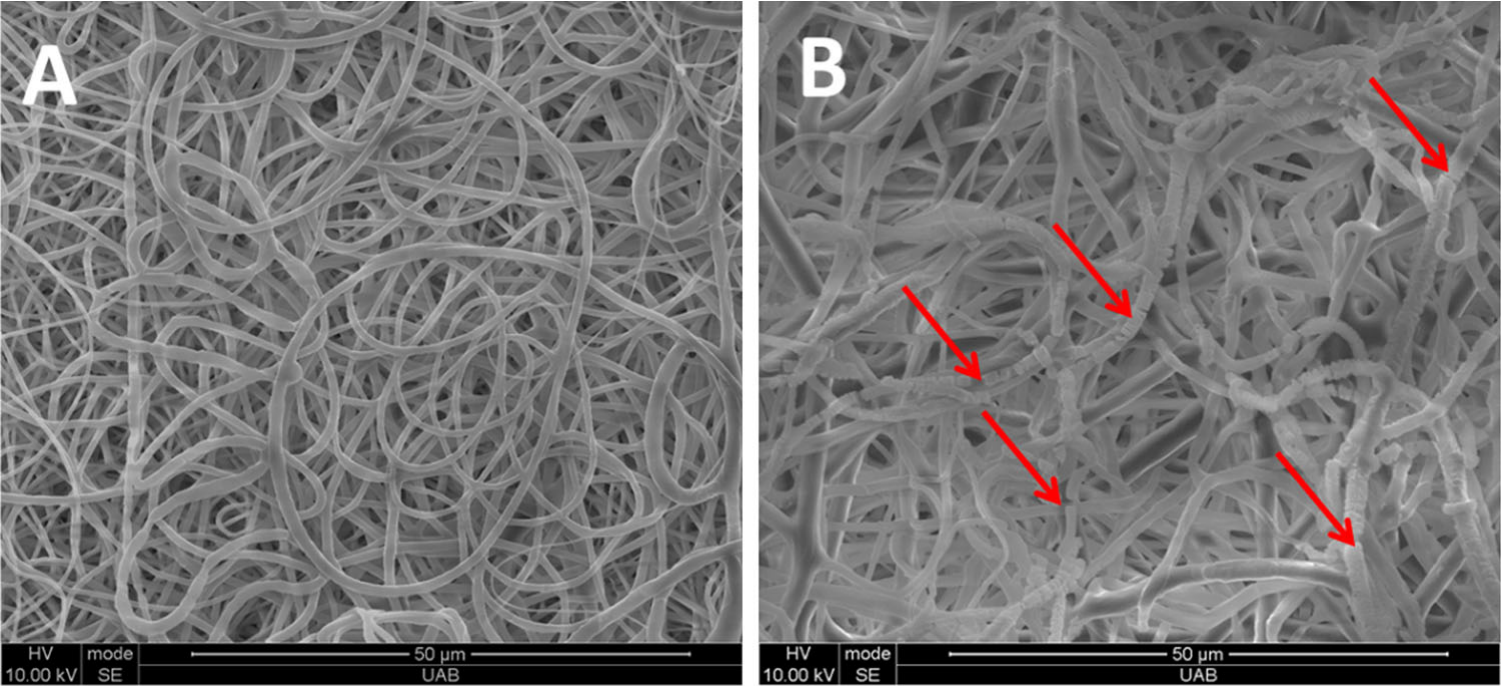
Lipase degradation effect on the fibers’ morphology **a** before and **b** after 4 weeks in lipase solution

The rate of the enzymatic degradation can be varied depending on the polymer type, crystallinity, and molecular weight (Nagata et al. 1998; Nikolic and Djonlagic 2001; Rizzarelli et al. 2004). Hence, the lipase enzymatic degradation effect on the mechanical properties as well as on morphology of the electrospun fibers were investigated for the electrospun graft made from the PCL:PGC blend. The specimens embedded in the lipase solution were taken out at 2 and 4 weeks’ time points and exposed to tensile tests. Tensile strength, Young’s modulus, and strain (%) data were obtained. The tensile strength showed a statistically significant (*p* < 0.05) decrease from 1.86 ± 0.14 to 1.49 ± 0.08 MPa after 4 weeks. The ultimate tensile strength even after 4 weeks was comparable to the human coronary artery values as reported by Holzapfel et al. (2005). As shown in Fig. 2b, no significant change in Young’s modulus was noticed after 4 weeks. The elastic modulus value after 4 weeks was found to be slightly higher than that reported by Ozolanta et al. (1998), which was around 4 MPa. On the other hand, the modulus of elasticity was lower than that of the native femoral artery (9–12 MPa) after 4 weeks’ degradation (Thomas et al. 2007). However, there was a significant (*p* < 0.05) difference in strain (%) before and after exposure to the lipase solution as shown in Fig. 2c. Figure 2d illustrates the mass loss (%) of the graft. The mass loss was increased significantly (*p* < 0.05) from 2 (3 ± 1 %) to 4 weeks (7.3 ± 2.6 %). This mass loss (%) also helps to correlate the decrease in tensile strength and stain (%) values. Figure 3a, b shows the electrospun fibers’ morphology before and after enzymatic degradation. Figure 3b clearly indicates the fiber brakeage due to the enzymatic degradation. Zeng et al. (2004) reported similar fiber breaking of PLLA–PCL based electrospun fibers.

ECM proteins coating on fibers of porous structure can facilitate cell attachment, migration, and infiltration. To incorporate the protein matrix, a simple dip-coating method was utilized (Peng et al. 2010). Zhang et al. (2005) also utilized the dip-coating method to increase surface biocompatibility of PCL non-woven scaffolds. Then, the presence of the protein matrix was confirmed by the DSC technique before and after aging in the media.

**Fig. 4:**
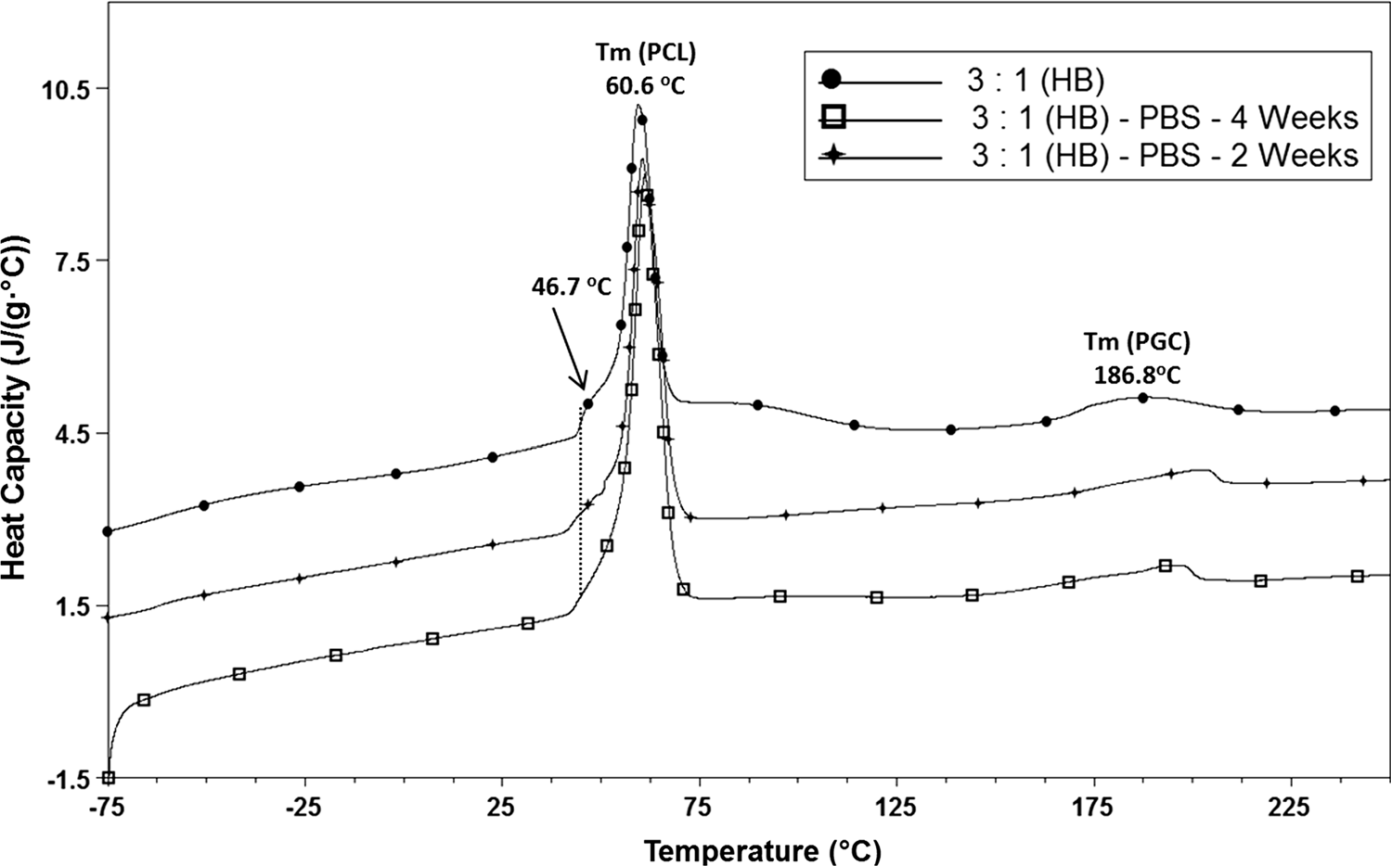
DSC curves of coated graft before and after in vitro environment exposure

**Fig. 5:**
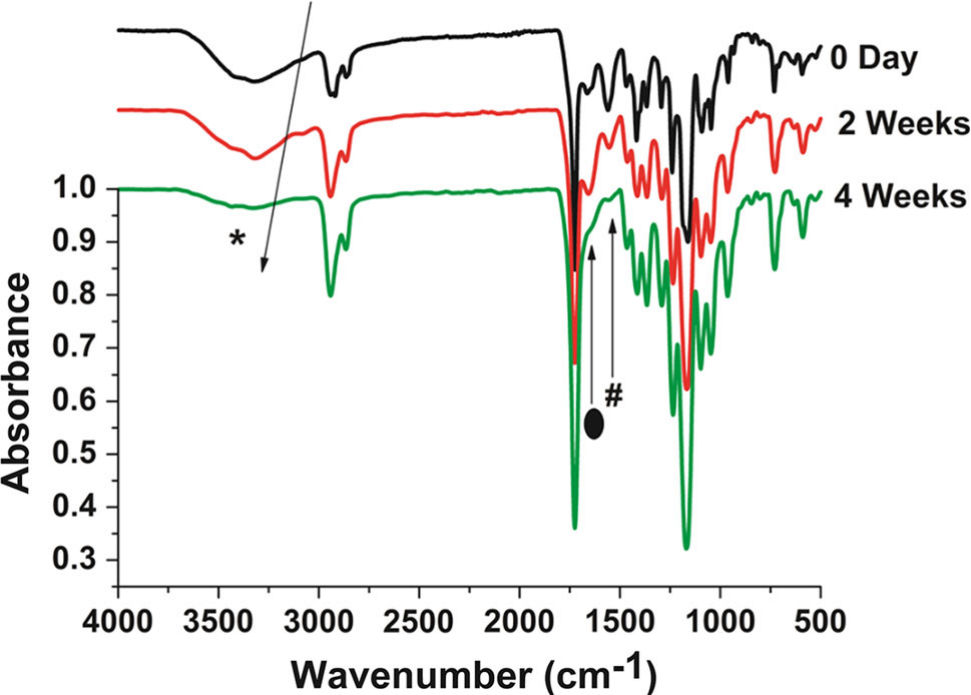
FTIR spectrum indicating protein matrix eroded after 4 weeks under in vitro condition. Proteins peaks were marked by symbols (*asterisk*, *hash*, *filled oval*)

DSC scans of 3:1 (PCL:PGC)-coated scaffold are shown in Fig. 4. The melting temperature of PCL was observed at 60.6 °C. Schindler et al. (2013) also observed a similar melting point for the PCL component of the PCL/polyglyconate blends. Further, Lee et al. (2008b) also mentioned a similar melting temperature (61.9 °C) for the PCL electrospun scaffold. The melting temperature of the PGC was noticed at 186.8 °C for 0 days. The shoulder peak at 46.7 °C could be an indication of the collagen helix (Mu et al. 2007). The shoulder around 46 °C started disappearing after 2 weeks and was absent at 4 weeks. This could be the indication that at 4 weeks’ time point, the protein matrix was not present in FTIR spectrum (Fig. 5).

The presence of N–H stretching peak at (*) 3310 cm^−1^ for amide A indicates the presence of protein from the protein matrix, since the non-coated graft does not have any amide group in the chemical structure. Further, amide I at 1651 cm^−1^ corresponds to the stretching vibration of the C=O bond. In addition, amide II at 1535 cm^−1^ is associated with the vibration of C–N and N–H bonds. Amide I and II were both present in the ATR spectrum of the protein matrix-coated graft. Jia et al. (2013) mentioned the presence of these peaks to identify the collagen component in the electrospun polyurethane-based scaffold. However, a clear decrease in the N–H stretching peak at 3310 cm^−1^ for amide A was noticed at 2 and 4 weeks’ time points. The decrease in the peak intensity may indicate the disappearance of the protein matrix. Similarly, the peak for amide I at 1651 cm^−1^ completely disappeared at 4 weeks’ time point. Zhang et al. (2009) also noticed a similar disappearance of the amine I peaks after 30 days due to the in vitro degradation of the bio-hybrid scaffold. This also indicates that the protein matrix was washed away in the PBS solution after 4 weeks. The data here suggest that the bonding between the protein matrix and synthetic polymer may be weak due to the non-covalent bonding, and covalent bonding may be required for a stable attachment of the protein matrix to the vascular graft.

**Fig. 6:**
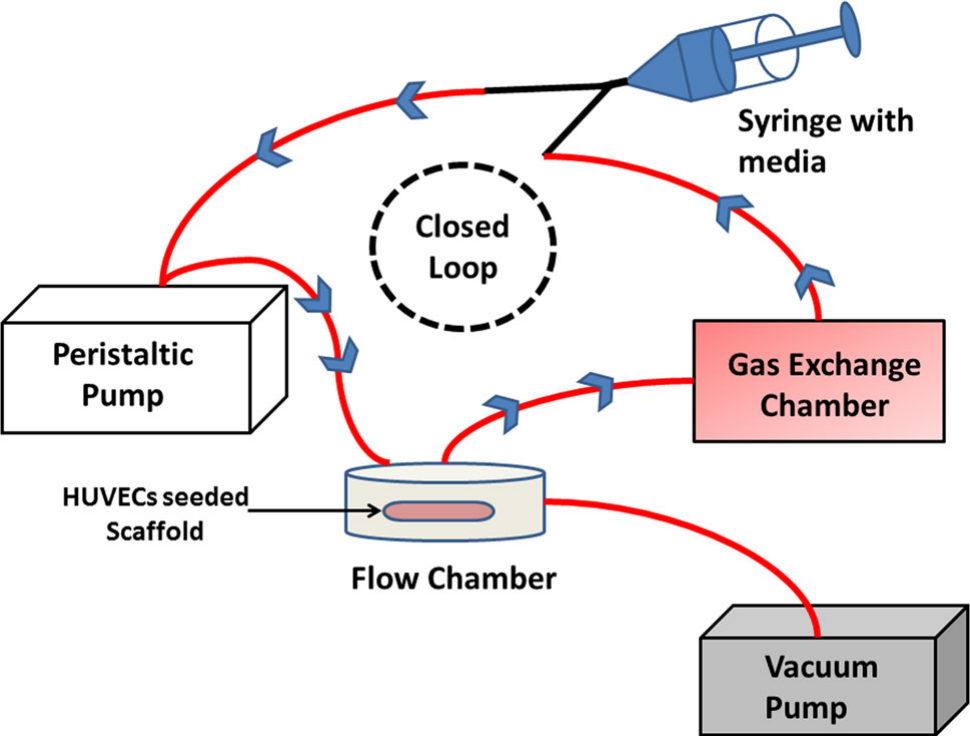
The setup of the dynamic flow chamber in the incubator

One of the goals of this study was to understand the cell attachment in static and dynamic conditions. In this study, we seeded HUVECs on the lumen surface of the coated graft. AoSMCs were seeded on the outer layer to understand their attachment on the coated graft. In a native blood vessel, endothelial cell which are present at a lumen surface experience a constant shear stress force due to blood flow which stimulates gene expression and effects cell metabolism as well as cell morphology (Traub and Berk 1998). Hence, the attachment of the HUVECs on our coated scaffolds under a shear stress environment is an important parameter to understand. As shown in Fig. 6, four critical components (Glyco-Tech flow chamber, a gasket, peristaltic pump, and a gas exchange chamber) were used. In order to calculate the flow rate to apply physiological shear stress we used Eq. 1 as mentioned below where *Q* is desired flow rate, *Τ* is the target shear stress, *µ* is the viscosity of the flow medium, *w* is width of the flow chamber, and *h* is the height of the flow chamber (Bhat et al. 1998).

1 $$ Q = \frac{{T\; \times \;w\; \times \; h^{2} }}{(6\; \times \;\mu )} $$

**Fig. 7:**
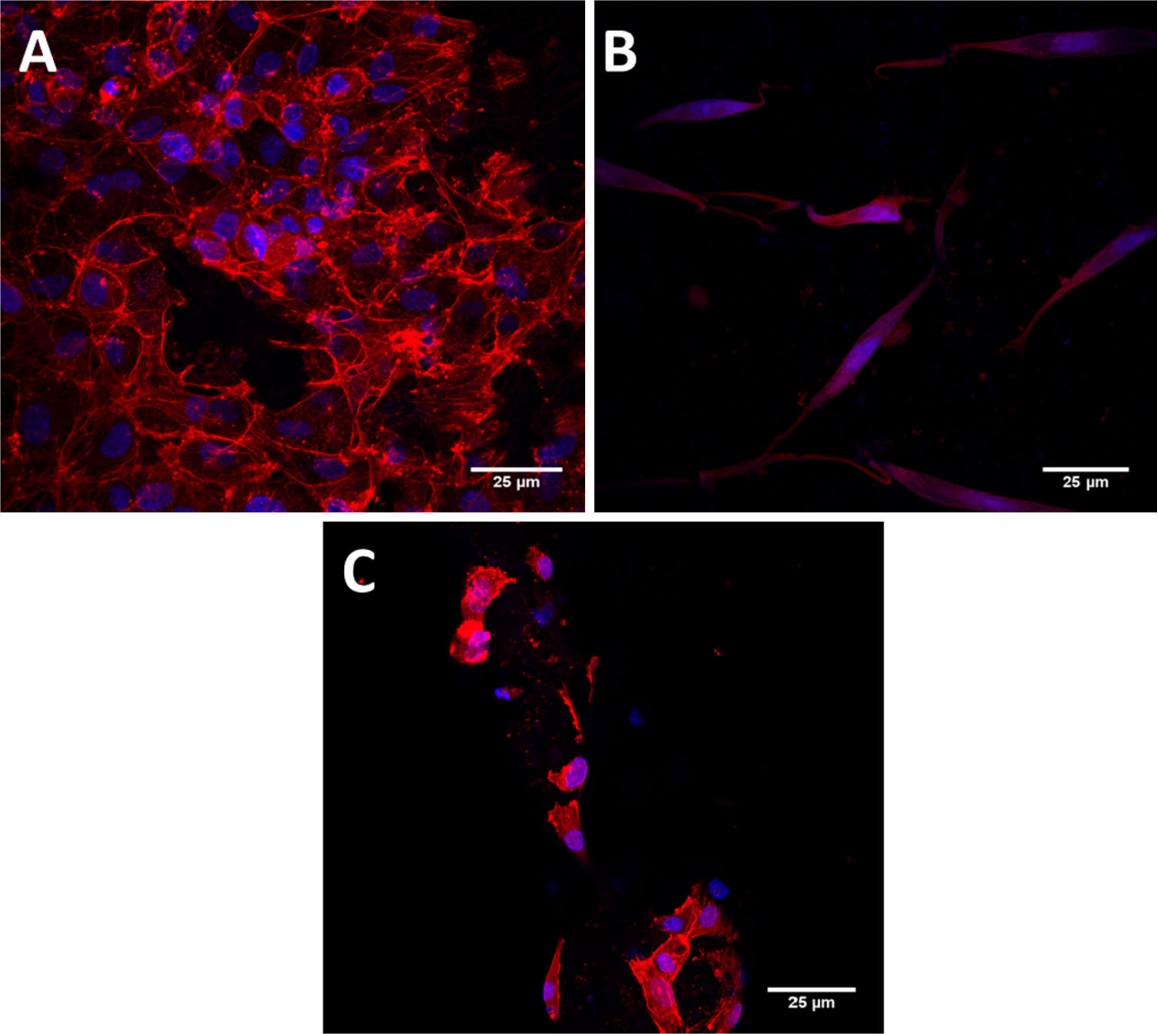
Confocal images of scaffolds seeded with **a** HUVECs and **b** AoSMCs. **c** HUVECs attachment after exposure to shear stress for 12 h

Figure 6 represents the entire closed loop system set up to understand the HUVECs attachment under the dynamic flow condition. Equation 1 was used to calculate the flow rate (8 mL/min) which was able to create a shear stress comparable to the physiological environment. Savoji et al. (2014) has also utilized similar technique to understand the stable endothelial spreading on the electrospun scaffolds under the shear stress. HUVECs were exposed to this shear stress continuously for 12 h. Then, HUVECs attachment on coated scaffold before and after exposure to laminar shear stress was observed by utilizing confocal microscopy. The comparison was done by qualitative analysis. Figure 7a represents a coated graft with HUVECs which was not exposed to a laminar flow as a control (Peng et al. 2010). Figure 7b represents a coated graft with AoSMCs which was in static condition. Figure 7c illustrates the HUVECs-seeded coated graft exposed to dynamic shear stress for 12 h. There was significantly fewer amount of cells present after laminar flow exposure compared to the control. The reason for fewer cells on the coated graft could be the weak non-covalent bonding between the fibers and protein matrix. Due to the weak bond, the protein matrix may have been washed out under shear stress, leading to elimination of the majority HUVECs. Hence, the protein matrix coating stabilization via a cross-linking agent may help a better cell attachment under shear stress environment.

## Conclusion

A small-diameter graft was engineered by utilizing the electrospinning technique. The graft was exposed to lipase enzymatic degradation for 1 month to understand the changes in mechanical properties. Significant changes in tensile strength (1.86–1.49 MPa) and strain (245–205 %) were noticed due to enzymatic degradation. The fiber breakage was clearly evident through scanning electron microscopy (SEM) after 4 weeks in vitro degradation. Further, the graft was coated with collagenous proteins and characterized by FTIR and DSC. A preliminary dynamic condition study to understand the HUVECs attachment was conducted. In static condition, HUVECs and AoSMCs attachment were observed. Under the dynamic flow condition, HUVECs attachment was found to be significantly lesser compared to the control (static condition). The weaker non-covalent bond between the protein matrix and the graft could be the reason. Further, the protein matrix eroded completely after 4 weeks in PBS solution, which was confirmed by DSC and FTIR. This issue will be addressed in a future study by introducing cross-linking agents to stabilize the protein matrix on the electrospun fibers.

## References

[CR1] Antunes J, Oliveira J, Reis R, Soria J, Gómez-Ribelles J, Mano J (2010). Novel poly (L-lactic acid)/hyaluronic acid macroporous hybrid scaffolds: characterization and assessment of cytotoxicity. J Biomed Mater Res Part A.

[CR2] Bezwada RS, Jamiolkowski DD, Lee I-Y, Agarwal V, Persivale J, Trenka-Benthin S, Erneta M, Suryadevara J, Yang A, Liu S (1995). Monocryl^®^ suture, a new ultra-pliable absorbable monofilament suture. Biomaterials.

[CR3] Bhat V, Truskey G, Reichert W (1998). Fibronectin and avidin–biotin as a heterogeneous ligand system for enhanced endothelial cell adhesion. J Biomed Mater Res.

[CR4] Boland ED, Coleman BD, Barnes CP, Simpson DG, Wnek GE, Bowlin GL (2005). Electrospinning polydioxanone for biomedical applications. Acta Biomater.

[CR5] Cittadella G, Mel A, Dee R, De Coppi P (2013). Seifalian am arterial tissue regeneration for pediatric applications: inspiration from up-to-date tissue-engineered vascular bypass grafts. Artif Organs.

[CR6] Clowes A, Gown A, Hanson S, Reidy M (1985). Mechanisms of arterial graft failure. 1. role of cellular proliferation in early healing of PTFE prostheses. Am J Pathol.

[CR7] Dahl SLM, Blum JL, LE Niklason (2011). Bioengineered vascular grafts: can we make them off-the-shelf?. Trends Cardiovasc Med.

[CR8] de Valence S, Tille JC, Mugnai D, Mrowczynski W, Gurny R, Moller M, Walpoth BH (2012). Long term performance of polycaprolactone vascular grafts in a rat abdominal aorta replacement model. Biomaterials.

[CR9] Dela Paz NG, D’Amore PA (2009). Arterial versus venous endothelial cells. Cell Tissue Res.

[CR10] Fowkes FG, Rudan D, Rudan I, Aboyans V, Denenberg JO, McDermott MM, Norman PE, Sampson UK, Williams LJ, Mensah GA, Criqui MH (2013). Comparison of global estimates of prevalence and risk factors for peripheral artery disease in 2000 and 2010: a systematic review and analysis. Lancet.

[CR11] Gan Z, Yu D, Zhong Z, Liang Q, Jing X (1999). Enzymatic degradation of poly (ε-caprolactone)/poly (DL-lactide) blends in phosphate buffer solution. Polymer.

[CR12] Gigout A, Ruiz JC, Wertheimer MR, Jolicoeur M, Lerouge S (2011). Nitrogen-rich plasma-polymerized coatings on pet and ptfe surfaces improve endothelial cell attachment and resistance to shear flow. Macromol Biosci.

[CR13] Go AS, Mozaffarian D, Roger VL, Benjamin EJ, Berry JD, Blaha MJ, Dai S, Ford ES, Fox CS, Franco S, Fullerton HJ, Gillespie C, Hailpern SM, Heit JA, Howard VJ, Huffman MD, Judd SE, Kissela BM, Kittner SJ, Lackland DT, Lichtman JH, Lisabeth LD, Mackey RH, Magid DJ, Marcus GM, Marelli A, Matchar DB, McGuire DK, Mohler ER, Moy CS, Mussolino ME, Neumar RW, Nichol G, Pandey DK, Paynter NP, Reeves MJ, Sorlie PD, Stein J, Towfighi A, Turan TN, Virani SS, Wong ND, Woo D, Turner MB (2014). Heart disease and stroke statistics–2014 update: a report from the American Heart Association. Circulation.

[CR14] Gunatillake PA, Adhikari R (2003). Biodegradable synthetic polymers for tissue engineering. Eur Cell Mater.

[CR15] Han J, Lazarovici P, Pomerantz C, Chen X, Wei Y, Lelkes PI (2010). Co-electrospun blends of PLGA, gelatin, and elastin as potential nonthrombogenic scaffolds for vascular tissue engineering. Biomacromolecules.

[CR16] He W, Ma Z, Yong T, Teo WE, Ramakrishna S (2005). Fabrication of collagen-coated biodegradable polymer nanofiber mesh and its potential for endothelial cells growth. Biomaterials.

[CR17] He W, Ma Z, Teo WE, Dong YX, Robless PA, Lim TC, Ramakrishna S (2009). Tubular nanofiber scaffolds for tissue engineered small-diameter vascular grafts. J Biomed Mater Res, Part A.

[CR18] He C, Xu X, Zhang F, Cao L, Feng W, Wang H, Mo X (2011). Fabrication of fibrinogen/P (LLA-CL) hybrid nanofibrous scaffold for potential soft tissue engineering applications. J Biomed Mater Res, Part A.

[CR19] Holzapfel GA, Sommer G, Gasser CT, Regitnig P (2005). Determination of layer-specific mechanical properties of human coronary arteries with nonatherosclerotic intimal thickening and related constitutive modeling. Am J Physiol Heart Circ Physiol.

[CR20] Jia L, Prabhakaran MP, Qin X, Kai D, Ramakrishna S (2013). Biocompatibility evaluation of protein-incorporated electrospun polyurethane-based scaffolds with smooth muscle cells for vascular tissue engineering. J Mater Sci.

[CR21] Ju YM, Choi JS, Atala A, Yoo JJ, Lee SJ (2010). Bilayered scaffold for engineering cellularized blood vessels. Biomaterials.

[CR22] Kidoaki S, Kwon IK, Matsuda T (2005). Mesoscopic spatial designs of nano- and microfiber meshes for tissue-engineering matrix and scaffold based on newly devised multilayering and mixing electrospinning techniques. Biomaterials.

[CR23] Lee K, Kim H, Khil M, Ra Y, Lee D (2003). Characterization of nano-structured poly (ε-caprolactone) nonwoven mats via electrospinning. Polymer.

[CR24] Lee SJ, Liu J, Oh SH, Soker S, Atala A, Yoo JJ (2008). Development of a composite vascular scaffolding system that withstands physiological vascular conditions. Biomaterials.

[CR25] Lee SJ, Oh SH, Liu J, Soker S, Atala A, Yoo JJ (2008). The use of thermal treatments to enhance the mechanical properties of electrospun poly (ε-caprolactone) scaffolds. Biomaterials.

[CR26] Liu H, Ding X, Zhou G, Li P, Wei X, Fan Y (2013). Electrospinning of nanofibers for tissue engineering applications. J Nanomater.

[CR27] Lu X, Sun Z, Cai W, Gao Z (2008). Study on the shape memory effects of poly (l-lactide-co-ε-caprolactone) biodegradable polymers. J Mater Sci Mater Med.

[CR28] Malek AM, Alper SL, Izumo S (1999). Hemodynamic shear stress and its role in atherosclerosis. JAMA.

[CR29] McClure M, Sell S, Ayres C, Simpson D, Bowlin G (2009). Electrospinning-aligned and random polydioxanone–polycaprolactone–silk fibroin-blended scaffolds: geometry for a vascular matrix. Biomed Mater.

[CR30] McClure MJ, Sell SA, Simpson DG, Bowlin GL (2009). Electrospun polydioxanone, elastin, and collagen vascular scaffolds: uniaxial cyclic distension. J Eng Fibers Fabr.

[CR31] Mu C, Li D, Lin W, Ding Y, Zhang G (2007). Temperature induced denaturation of collagen in acidic solution. Biopolymers.

[CR32] Nagata M, Machida T, Sakai W, Tsutsumi N (1998). Synthesis, characterization, and enzymatic degradation studies on novel network aliphatic polyesters. Macromolecules.

[CR33] Nikolic MS, Djonlagic J (2001). Synthesis and characterization of biodegradable poly (butylene succinate-co-butylene adipate) s. Polym Degrad Stab.

[CR34] Ozolanta I, Tetere G, Purinya B, Kasyanov V (1998). Changes in the mechanical properties, biochemical contents and wall structure of the human coronary arteries with age and sex. Med Eng Phys.

[CR35] Pandis L, Zavan B, Abatangelo G, Lepidi S, Cortivo R, Vindigni V (2010). Hyaluronan-based scaffold for in vivo regeneration of the rat vena cava: preliminary results in an animal model. J Biomed Mater Res Part A.

[CR36] Pankajakshan D, Philipose LP, Palakkal M, Krishnan K, Krishnan LK (2008). Development of a fibrin composite-coated poly (ε-caprolactone) scaffold for potential vascular tissue engineering applications. J Biomed Mater Res B Appl Biomater.

[CR37] Patel HN, Garcia R, Schindler C, Dean D, Pogwizd SM, Singh R, Vohra YK, Thomas V (2015). Fibro‐porous poliglecaprone/polycaprolactone conduits: synergistic effect of composition and in vitro degradation on mechanical properties. Polym Int.

[CR38] Peña J, Corrales T, Izquierdo-Barba I, Doadrio AL, Vallet-Regí M (2006). Long term degradation of poly (ε-caprolactone) films in biologically related fluids. Polym Degrad Stab.

[CR39] Peng H, Ling J, Liu J, Zhu N, Ni X, Shen Z (2010). Controlled enzymatic degradation of poly (ε-caprolactone)-based copolymers in the presence of porcine pancreatic lipase. Polym Degrad Stab.

[CR40] Rizzarelli P, Impallomeni G, Montaudo G (2004). Evidence for selective hydrolysis of aliphatic copolyesters induced by lipase catalysis. Biomacromolecules.

[CR41] Savoji H, Hadjizadeh A, Maire M, Ajji A, Wertheimer MR, Lerouge S (2014). Electrospun nanofiber scaffolds and plasma polymerization: a promising combination towards complete, stable endothelial lining for vascular grafts. Macromol Biosci.

[CR42] Schindler C, Williams BL, Patel HN, Thomas V, Dean DR (2013). Electrospun polycaprolactone/polyglyconate blends: miscibility, mechanical behavior, and degradation. Polymer.

[CR43] Seifu DG, Purnama A, Mequanint K, Mantovani D (2013). Small-diameter vascular tissue engineering. Nat Rev Cardiol.

[CR44] Sell SA, McClure MJ, Garg K, Wolfe PS, Bowlin GL (2009). Electrospinning of collagen/biopolymers for regenerative medicine and cardiovascular tissue engineering. Adv Drug Deliv Rev.

[CR45] Sell SA, Wolfe PS, Garg K, McCool JM, Rodriguez IA, Bowlin GL (2010). The use of natural polymers in tissue engineering: a focus on electrospun extracellular matrix analogues. Polymers.

[CR46] Siegal GP, Singh R (2010) Biologically active native biomatrix composition. US Patent 7,727,550

[CR47] Song Y, Feijen J, Grijpma D, Poot A (2011). Tissue engineering of small-diameter vascular grafts: a literature review. Clinical Hemorheol Microcirc.

[CR48] Stegemann JP, Kaszuba SN, Rowe SL (2007). Review: advances in vascular tissue engineering using protein-based biomaterials. Tissue Eng.

[CR49] Stitzel JD, Pawlowski KJ, Wnek GE, Simpson DG, Bowlin GL (2001). Arterial smooth muscle cell proliferation on a novel biomimicking, biodegradable vascular graft scaffold. J Biomater Appl.

[CR50] Thomas V, Zhang X, Catledge SA, Vohra YK (2007). Functionally graded electrospun scaffolds with tunable mechanical properties for vascular tissue regeneration. Biomed Mater.

[CR51] Tokiwa Y, Suzuki T (1977). Hydrolysis of polyesters by lipases. Nature.

[CR52] Traub O, Berk BC (1998). Laminar shear stress mechanisms by which endothelial cells transduce an atheroprotective force. Arterioscler Thromb Vasc Biol.

[CR53] Wang X, Lin P, Yao Q, Chen C (2007). Development of small-diameter vascular grafts. World J Surg.

[CR54] Wang S, Zhang Y, Yin G, Wang H, Dong Z (2009). Electrospun polylactide/silk fibroin–gelatin composite tubular scaffolds for small-diameter tissue engineering blood vessels. J Appl Polym Sci.

[CR55] Yin A, Zhang K, McClure MJ, Huang C, Wu X, Fang J, Mo X, Bowlin Q, Al-Deyab X, Wu X (2013). Electrospinning collagen/chitosan/poly (L-lactic acid-co-ε-caprolactone) to form a vascular graft: mechanical and biological characterization. J Biomed Mater Res Part A.

[CR56] Zeng J, Chen X, Liang Q, Xu X, Jing X (2004). Enzymatic degradation of poly (L-lactide) and poly (ε-caprolactone) electrospun fibers. Macromol Biosci.

[CR57] Zhang Y, Venugopal J, Huang Z-M, Lim C, Ramakrishna S (2005). Characterization of the surface biocompatibility of the electrospun PCL-collagen nanofibers using fibroblasts. Biomacromolecules.

[CR58] Zhang X, Thomas V, Vohra YK (2009). *In vitro* biodegradation of designed tubular scaffolds of electrospun protein/polyglyconate blend fibers. J Biomed Mater Res B Appl Biomater.

[CR59] Zhang X, Thomas V, Xu Y, Bellis SL, Vohra YK (2010). An in vitro regenerated functional human endothelium on a nanofibrous electrospun scaffold. Biomaterials.

